# Supplementary figures and images for: Correlated Transcriptional Responses Provide Insights into the Synergy Mechanisms of the Furazolidone, Vancomycin, and Sodium Deoxycholate Triple Combination in Escherichia coli
Source: mSphere. 2021 Sep 8;6(5):e00627-21. doi: 10.1128/mSphere.00627-21 (PMC8550143; doi:10.1128/mSphere.00627-21)

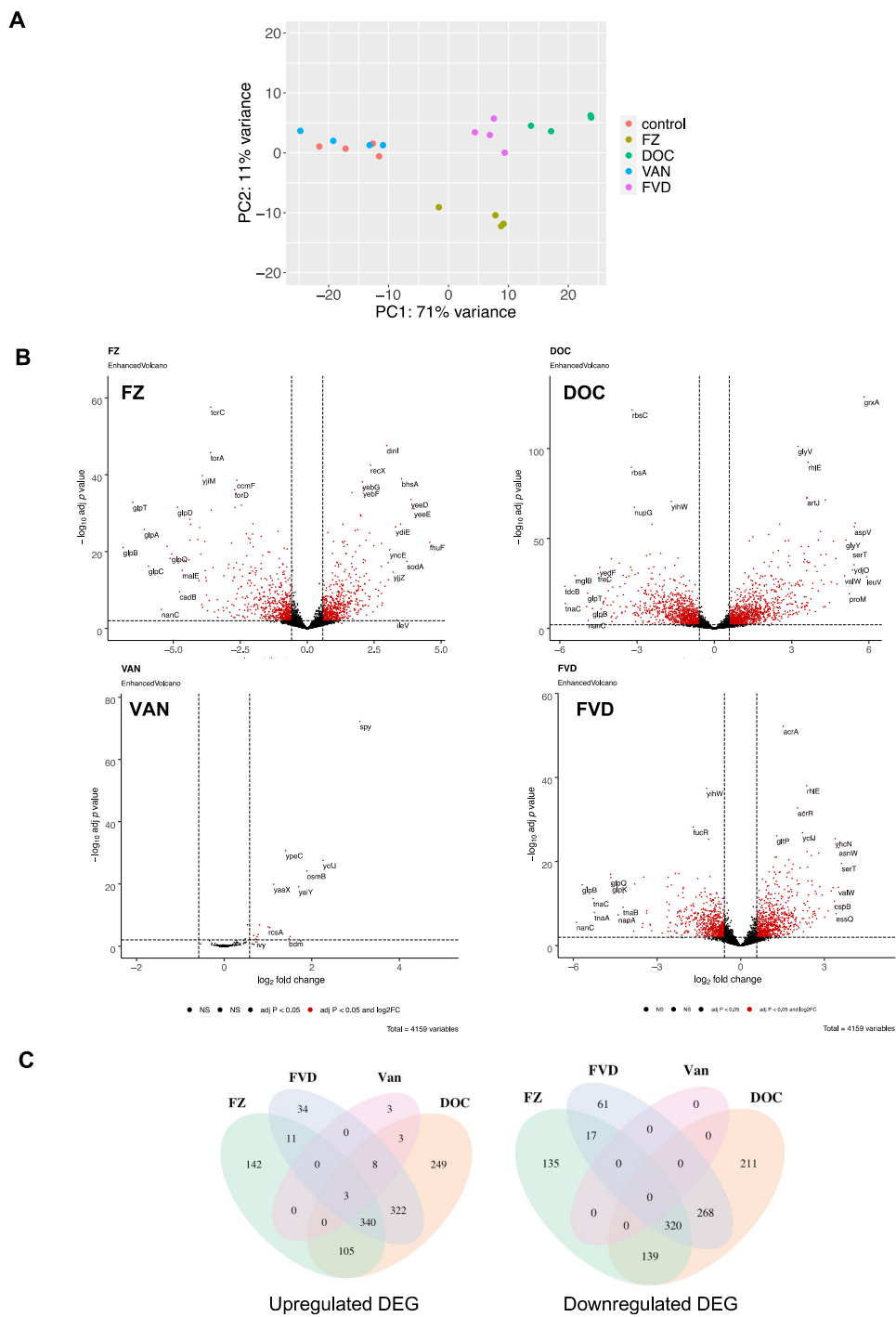

**Fig. S1**

Supplement: FIG S1 [file msphere.00627-21-sf001.pdf]

**A**

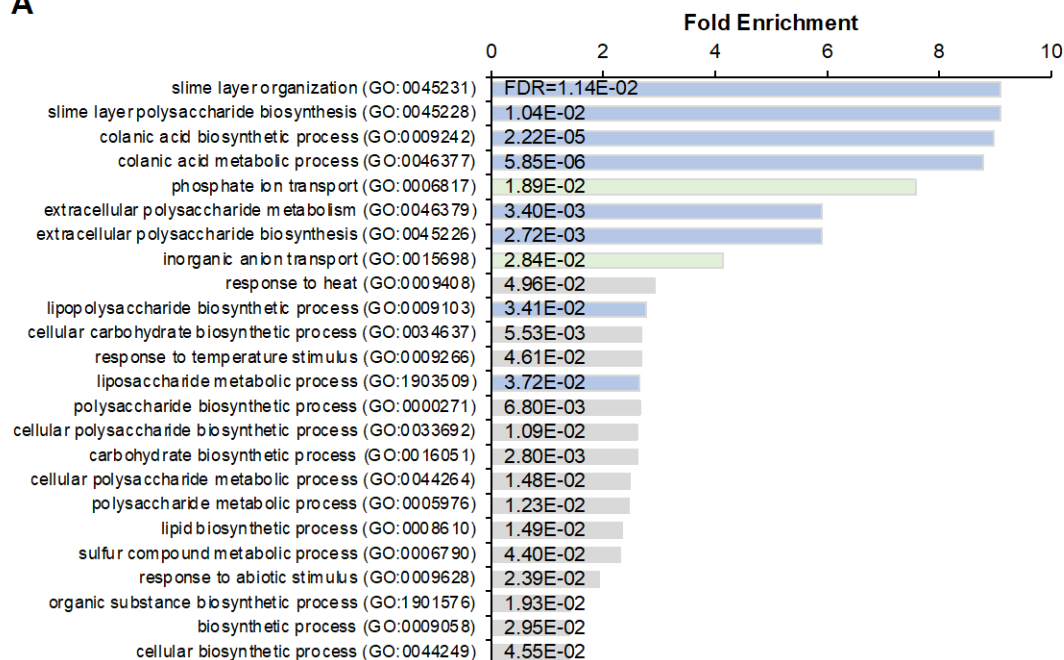

**B**

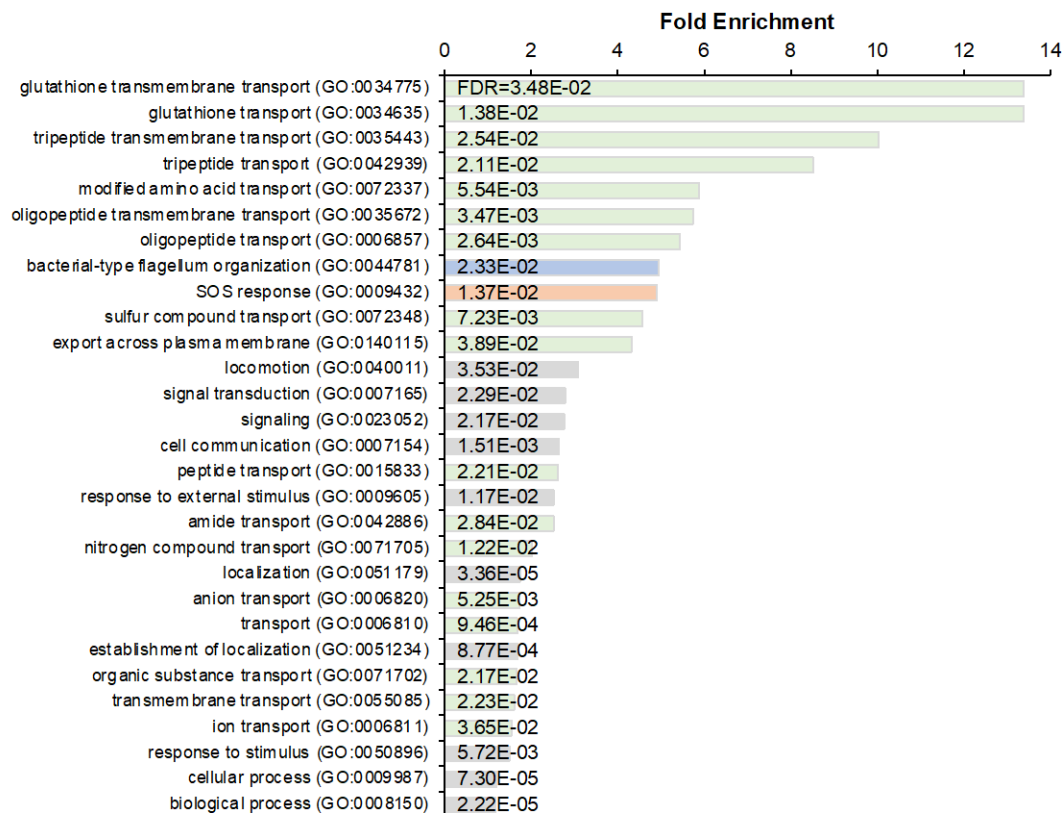

**Fig. S2**

Supplement: FIG S2 [file msphere.00627-21-sf002.pdf]

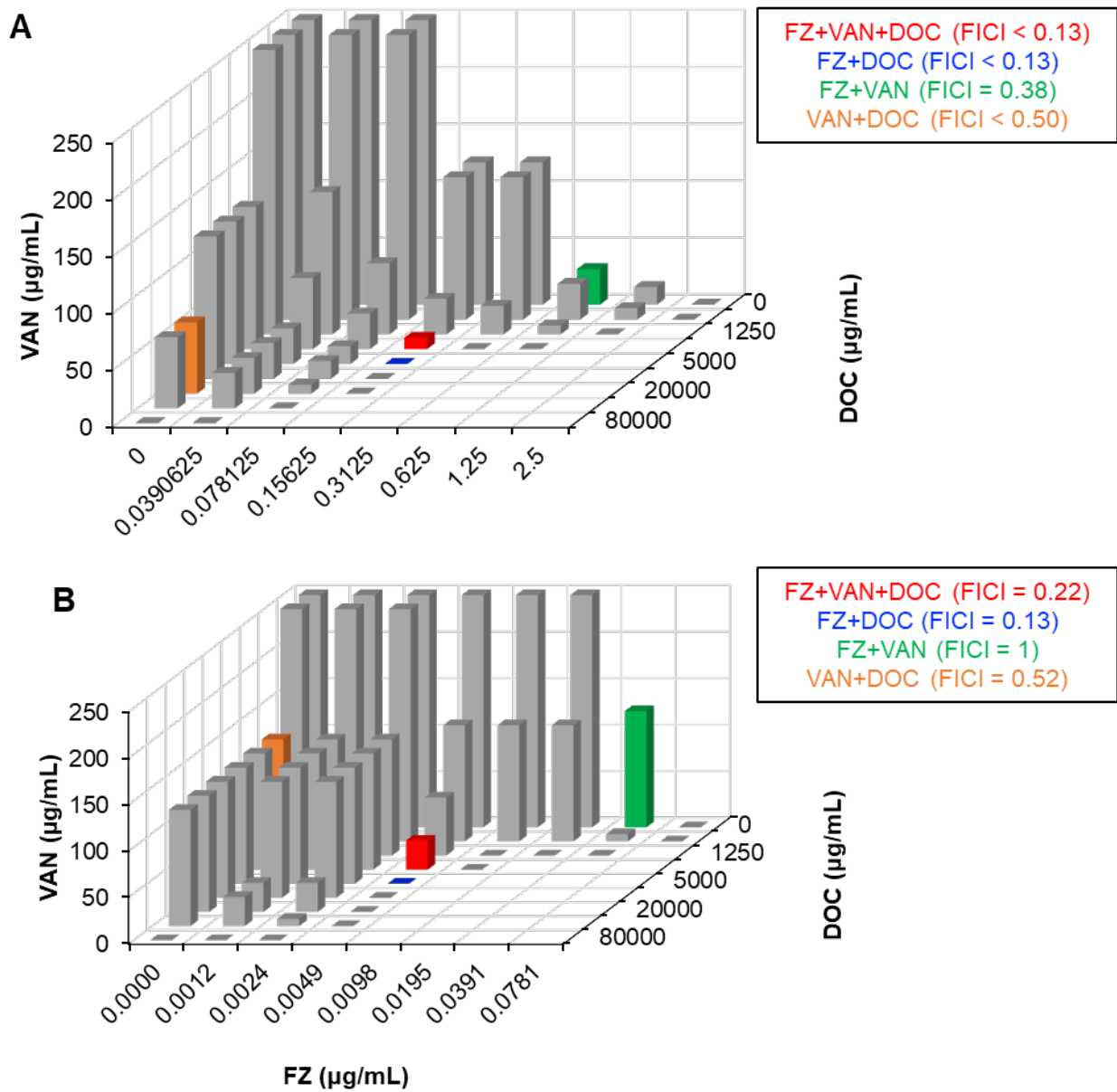

**Fig. S3**

Supplement: FIG S3 [file msphere.00627-21-sf003.pdf]
