# Supplementary material for: Correlated Transcriptional Responses Provide Insights into the Synergy Mechanisms of the Furazolidone, Vancomycin, and Sodium Deoxycholate Triple Combination in Escherichia coli
Source: mSphere. 2021 Sep 8;6(5):e00627-21. doi: 10.1128/mSphere.00627-21 (PMC8550143; doi:10.1128/mSphere.00627-21)
Supplement: TABLE S1 [file msphere.00627-21-st001.pdf]

Table S1

		Concentration (µg/mL)		
**Sample name**	**Treatment**	**FZ**	**DOC**	**VAN**
control	0.1 DMSO	0	0	0
FZ	FZ IC_{50}	1.9	0	0
DOC	DOC IC_{50}	0	5000	0
VAN	VAN IC_{50}	0	0	190
FVD	FZ+DOC+VAN IC_{50}	0.117	1875	7.5
