# Supplementary material for: Correlated Transcriptional Responses Provide Insights into the Synergy Mechanisms of the Furazolidone, Vancomycin, and Sodium Deoxycholate Triple Combination in Escherichia coli
Source: mSphere. 2021 Sep 8;6(5):e00627-21. doi: 10.1128/mSphere.00627-21 (PMC8550143; doi:10.1128/mSphere.00627-21)
Supplement: TABLE S2 [file msphere.00627-21-st002.pdf]

Table S2

Sample	Total Reads	Error Rate[a] (%)	Q20[b] (%)	Q30[b] (%)	GC content (%)	Uniquely aligned reads (% of total)	Multi-aligned reads (% of total)	Unaligned reads (% of total)	SRA Accession
control 1	29407278	0.03	98.19	94.47	52.45	96.62	1.62	1.77	SRX8634415
control 2	22854368	0.02	98.23	94.65	52.47	92.31	1.94	5.76	SRX8634416
control 3	24939548	0.03	98.03	94.19	52.17	96.62	0.88	2.50	SRX8634419
control 4	25423669	0.02	98.25	94.69	52.23	96.70	1.04	2.26	SRX8634420
FZ 1	21921743	0.02	98.15	94.44	52.04	96.83	1.07	2.10	SRX8634421
FZ 2	24047820	0.03	98.09	94.24	51.97	96.76	1.10	2.13	SRX8634422
FZ 3	23367043	0.03	98.11	94.37	52.17	96.81	1.05	2.14	SRX8634423
FZ 4	24157475	0.03	98.12	94.34	52.13	96.54	1.10	2.36	SRX8634424
DOC 1	25720688	0.02	98.21	94.59	51.53	95.25	1.17	3.58	SRX8634425
DOC 2	22741056	0.03	98.04	94.19	51.39	96.12	0.93	2.95	SRX8634426
DOC 3	25073567	0.02	98.22	94.61	51.67	96.73	0.99	2.29	SRX8634417
DOC 4	20296896	0.02	98.22	94.59	51.75	95.97	0.96	3.07	SRX8634418
VAN 1	21527873	0.03	97.88	93.84	52.21	95.99	1.55	2.46	SRX9524543
VAN 2	26529764	0.02	98.29	94.78	52.51	96.97	1.21	1.82	SRX9524544
VAN 3	29217877	0.02	98.22	94.63	52.47	97.27	0.99	1.73	SRX9524545
VAN 4	29779583	0.02	98.21	94.61	52.34	96.95	1.00	2.05	SRX9524546
FVD 1	25265808	0.02	98.17	94.52	51.93	97.19	0.95	1.86	SRX9530092
FVD 2	21078348	0.03	97.95	94.01	52.06	96.10	1.02	2.88	SRX9530093
FVD 3	20244157	0.03	97.87	93.85	52.06	96.92	1.06	2.02	SRX9530094
FVD 4	22324581	0.03	98.01	94.15	51.85	97.03	0.97	2.00	SRX9530095

[a]Error rate: base error rate

[b]Q20, Q30: (Base count of Phred value > 20 or 30) / (Total base count)
